# Global metabolomics reveals potential urinary biomarkers of esophageal squamous cell carcinoma for diagnosis and staging

**DOI:** 10.1038/srep35010

**Published:** 2016-10-11

**Authors:** Jing Xu, Yanhua Chen, Ruiping Zhang, Jiuming He, Yongmei Song, Jingbo Wang, Huiqing Wang, Luhua Wang, Qimin Zhan, Zeper Abliz

**Affiliations:** 1 State Key Laboratory of Bioactive Substance and Function of Natural Medicines, Institute of Materia Medica, Chinese Academy of Medical Sciences & Peking Union Medical College, Beijing 100050, P. R. China; 2 State Key Laboratory of Molecular Oncology, Cancer Institute & Hospital, Chinese Academy of Medical Sciences & Peking Union Medical College, Beijing 100021, P. R. China; 3 Department of Radiation Oncology, Cancer Institute & Hospital, Chinese Academy of Medical Sciences & Peking Union Medical College, Beijing 100021, P. R. China; 4 Centre for Bioimaging & Systems Biology, Minzu university of China, Beijing 100081, P. R. China

## Abstract

We performed a metabolomics study using liquid chromatography-mass spectrometry (LC-MS) combined with multivariate data analysis (MVDA) to discriminate global urine profiles in urine samples from esophageal squamous cell carcinoma (ESCC) patients and healthy controls (NC). Our work evaluated the feasibility of employing urine metabolomics for the diagnosis and staging of ESCC. The satisfactory classification between the healthy controls and ESCC patients was obtained using the MVDA model, and obvious classification of early-stage and advanced-stage patients was also observed. The results suggest that the combination of LC-MS analysis and MVDA may have potential applications for ESCC diagnosis and staging. We then conducted LC-MS/MS experiments to identify the potential biomarkers with large contributions to the discrimination. A total of 83 potential diagnostic biomarkers for ESCC were screened out, and 19 potential biomarkers were identified; the variations between the differences in staging using these potential biomarkers were further analyzed. These biomarkers may not be unique to ESCCs, but instead result from any malignant disease. To further elucidate the pathophysiology of ESCC, we studied related metabolic pathways and found that ESCC is associated with perturbations of fatty acid β-oxidation and the metabolism of amino acids, purines, and pyrimidines.

Esophageal cancer (EC) is a common cause of cancer-related death. In 2012, an estimated 455,800 new cases of EC were observed worldwide, and 400,200 deaths occurred as a result of EC^1^. China is a high-risk area for EC, and more than 90% of cases are esophageal squamous cell carcinoma (ESCC)^2^,^3^. Early-stage asymptomatic EC is usually curable with excellent long-term survival (90% or above at 5 years). Unfortunately, most patients exhibit locally advanced or metastatic EC at the time of diagnosis and have a poor prognosis (5-year survival rate, <20%)^4^,^5^,^6^. Even among patients with radical esophagectomies, the 5-year survival rate is only 10–20%^7^. Clearly, the discovery of potential biomarkers for early diagnosis of EC are urgently needed.

The advent of EC is accompanied by metabolic changes that are reflected by changes in gene expression, microRNA profiles, and the concentrations of circulating proteins and small metabolites^8^,^9^. Metabolomics involves the global and unbiased definition of the complement of small molecules in biofluids, tissues, organs, or organisms^10^,^11^,^12^,^13^. Therefore, this approach facilitates the screening and early detection of EC. Because metabolomics can provide information about disease processes, this method has been widely applied to the diagnosis of multiple pathologies^14^,^15^,^16^,^17^. Currently, nuclear magnetic resonance (NMR) spectroscopy and mass spectrometry (MS) are the most widely employed methods for metabolomics studies^18^. Compared with NMR, MS (particularly liquid chromatography [LC]-MS) offers several advantages, including high sensitivity and a wide dynamic range, and thus, LC-MS has become increasingly popular for metabolomics studies in recent years^19^,^20^,^21^,^22^.

Several metabolomics studies of EC have been performed using various analytical platforms^23^,^24^,^25^,^26^. Previously, we also performed global and targeted metabolomics study of ESCC plasma to discover potential diagnostic and therapeutic biomarkers^27^. Compared to plasma, urine is more readily available and is noninvasively collected. Moreover, urine is not subject to homeostatic mechanisms, and greater varieties of endogenous metabolites could occur in urine, thereby better reflecting the changes in human metabolism.

Here, we performed LC-MS combined with multivariate data analysis (MVDA) to investigate the global urinary profiles of ESCC patients and normal controls. In addition, we evaluated the possibility of using urine metabolomics for the classification of ESCC and used an independent test set to examine the predictive ability of the analytical platform. Potential biomarkers were discovered, identified, and evaluated by receiver operating characteristic analysis (ROC). Moreover, we monitored the variation in these biomarkers during staging. Compare with the plasma metabolomics results, we investigated related metabolic pathways. The overall goals of this study were to (1) develop a LC-MS-based urine metabolomics method for ESCC diagnosis and staging, (2) discover potential biomarkers, and (3) illustrate the pathological changes associated with ESCC. The workflow of this study is shown in Figure S1.

## Results

### Data quality assessment

To obtain reliable data from metabolomics analysis, using a stable analytical method is important. Accordingly, unsupervised principal component analysis (PCA) was performed on all samples (including ESCC patients, NC and quality control (QC) samples) as part of an assessment of the stability of the process. Figure S2 shows that all QCs clustered in the center of the PCA. This results demonstrated no drift in retention time and chromatographic shape during the whole run-sequence. indicating the LC-MS results were statistically acceptable for analysis^28^,^29^. Furthermore, the mixed standard was also analyzed simultaneously with the acquired samples to evaluate the reproducibility of the method. The extracted ion chromatograms (XICs) of the mixed standard are shown in Figure S3. The relative standard deviations of the retention times of each standard compound in both ion modes were less than 5%, and the relative standard deviations of peak areas were below 15% (Table 1). In addition, the retention time deviation profiles of all urine samples resulted from R-software exhibited ±20 s fluctuation in most of the LC-(±) electrospray ionization (ESI)-MS analyses (Figure S4). These results indicated that chromatographic separation and mass measurement were highly stable and reproducible throughout the sequence. The typical total ion chromatograms (TICs) produced from urine samples by LC-(±)ESI-MS are presented in Figure S5.

### Multivariate statistical analysis

The LC-ESI-MS data sets obtained in both positive- and negative-ion modes contained 1463 and 2153 peaks, respectively, with retention times of 0.9–25 min. The program coded for LC-(±)ESI-MS analysis is available in the Supporting Information. Unsupervised PCA by SIMCA-P was initially conducted to obtain an overview of the urinary LC-MS data from cancer patients and healthy controls. As shown in Figure S6, obvious separation trends between the two groups indicated that the ESCC patients exhibited metabolic changes relative to those of the controls.

**Figure 1:**
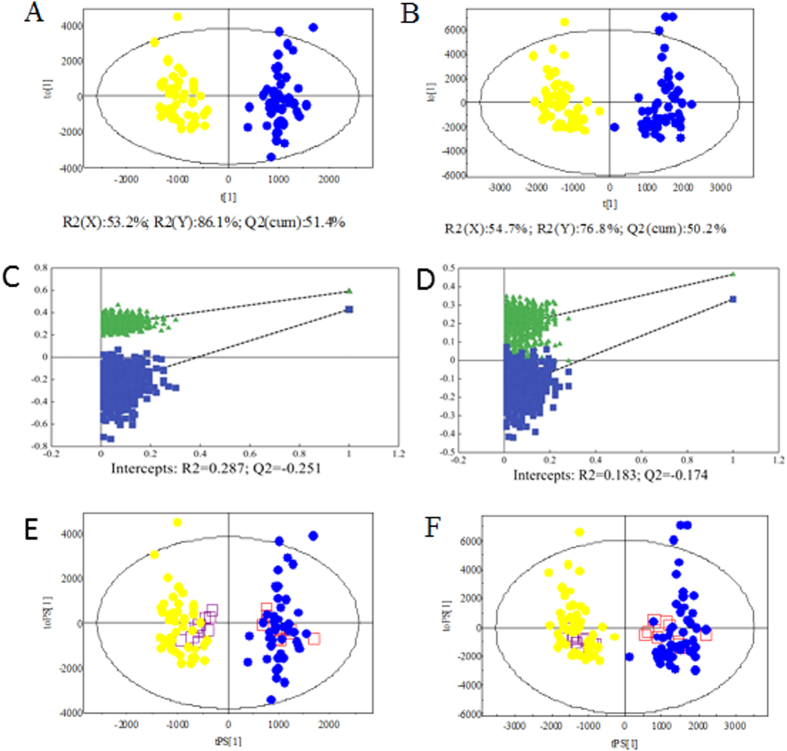
Score plots of OPLS-DA models (**A,B**); Validation plots of the PLS-DA models (**C,D**) and T-predicted scatter plots (**E,F**) of OPLS-DA model based on the data from LC-(+)ESI-MS (**A,C,E**) and LC-(−)ESI-MS (**B,D,F**). (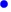, ESCC patients; 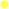, controls; 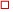, ESCC patient prediction set; and 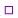, control prediction set).

To maximize the discrimination among the classes of observations and explore potential biomarker candidates in more detail, we applied orthogonal partial least squares discriminant analysis (OPLS-DA) as a stoichiometric analysis method to explore the difference between patients and controls. Ten ESCC patients and 10 controls were randomly selected to form an independent test set, and the training set consisted of the remaining subjects. The score scatter plots for the LC-(±)ESI-MS data of the training set showed clear discrimination between the ESCC and NC groups (Fig. 1A,B). For LC-(+)ESI-MS data, the one predictive (t_p_) and two orthogonal (t_o_) (1 + 2) components were calculated with *R*^*2*^(Y) and *Q*^*2*^(cum) values of 86.1% and 51.4%, respectively. The LC-(−)ESI-MS data set generated one predictive and three orthogonal components with *R*^*2*^(Y) and *Q*^*2*^(cum) values of 76.8% and 50.2%, respectively.

To prevent original model overfitting, permutation tests with 999 iterations were performed (Fig. 1C,D). These permutation tests produced intercepts of *R*^*2*^ and *Q*^*2*^ with values of 0.287 and −0.251 for the positive model data and 0.183 and −0.174 for the negative model data (Fig. 1C,D). The criteria for validity were as follows: *R*^*2*^ less than 0.4, and *Q*^*2*^ less than 0.05^30^. Thus, the results indicate that the OPLS-DA models generated from the LC-(±)ESI-MS data were reliable.

To further evaluate the predictive ability of the established models, an external test using plasma samples from 10 patients and 10 controls was performed. As shown in Fig. 1E,F, satisfactory results were obtained. The OPLS-DA model correctly predicted all ESCC patients and healthy controls with 100% sensitivity and specificity while 1463 and 2153 ions of interest were applied. This result indicated that LC-MS-based urine metabolomics has potential applications for non-invasive ESCC diagnosis.

**Figure 2:**
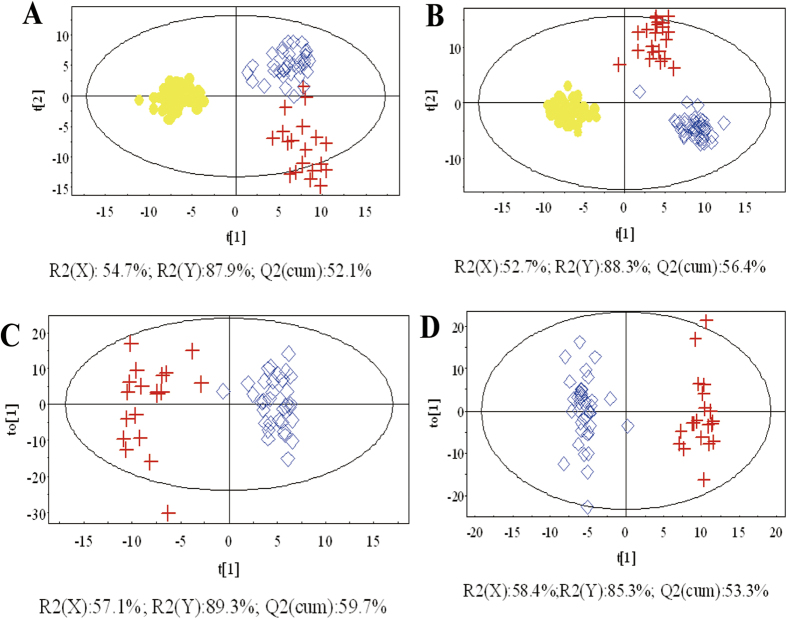
OPLS-DA score plots based on the data from (**A,C**) LC-(+)ESI-MS and (**B,D**) LC-(−)ESI-MS of ESCC patients and healthy controls. 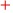, early-stage ESCC patients (T1–2); 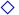, advanced-stage ESCC patients (T3–4); and 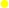, controls.

Furthermore, OPLS-DA was applied to differentiate early-stage (T1–2) and advanced-stage (T3–4) ESCC patients. The score scatter plots of OPLS-DA models from the LC-(±)ESI-MS data showed the clear differentiation of early-stage (T1–2) ESCC patients, advanced-stage (T3–4) ESCC patients, and NC groups (Fig. 2), indicating that changes in some endogenous metabolites were related to disease stage. The results of permutation tests with 999 iterations showed that the models were not overfitted (Figure S7).

### Discovery, identification and characterization of potential biomarkers

Based on the OPLS-DA results, which facilitated a good group classification of ESCC patients and controls, we extracted potential markers of interest from the combined S-plot, variable importance in project (VIP), and raw data plots^31^,^32^. An independent t-test (*P* < 0.05) was also performed to validate the significance of the discriminated variables selected by these methods. XICs were used to reduce the redundant variables originating from the same compound, such as adduct ions, fragments and isotopes. Ultimately, we selected 83 biomarker candidates for further identification.

**Figure 3:**
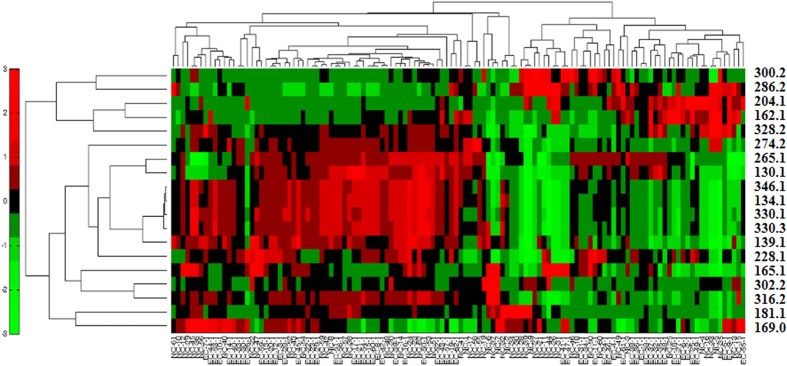
Hierarchical Clustering Analysis (HCA) of 19 potential biomarkers.

The possible elemental compositions of the biomarkers were determined based on their exact masses, considering the relative intensities of the isotope peaks on high-resolution MS spectra. Furthermore, we elucidated the structures of the potential biomarkers based on high-resolution MS/MS spectra and searches in various databases. Subsequently, standard compounds were used to confirm the structures of these metabolites. Ultimately, 19 potential diagnostic biomarkers were identified, including nine carnitine derivatives (L-carnitine and eight acylcarnitines), four amino acid derivatives (pyroglutamic acid, indoxyl, urocanic acid, and phenylacetylglutamine), three nucleosides (deoxycytidine, cyclic adenosine monophosphate [cAMP], and cyclic guanosine monophosphate [cGMP]), two purine derivatives (uric acid and paraxanthine), and L-Fucose. Detailed information regarding these compounds is listed in Table 2 and Figures S8–S25. Nine were further confirmed by comparison with authentic standards, including retention times and MS/MS fragmentation patterns. The identification score is also calculated by the scoring metric^33^. Hierarchical clustering analysis (HCA) of these potential biomarkers was conducted, and the results are shown in Fig. 3.

**Figure 4:**
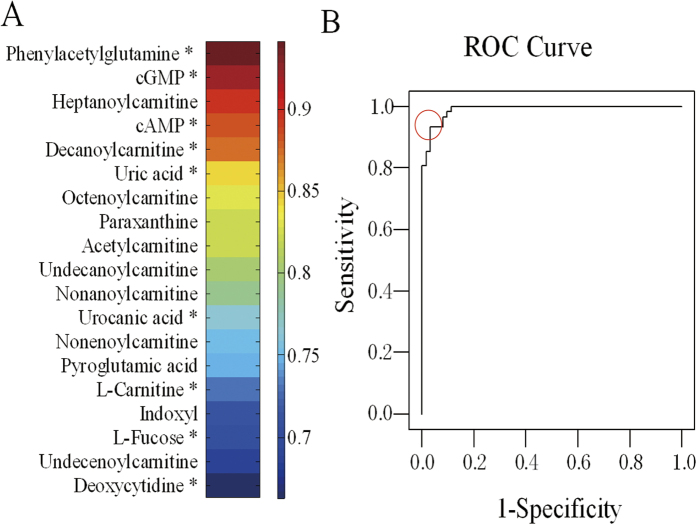
Visualization of the discriminatory power of individual and combined potential diagnostic biomarkers.

To further characterize the utility of these potential biomarkers for the prediction of ESCC, univariate ROC analysis and heat maps were carried out. The ROC curve could provide information regarding the sensitivity and specificity of the potential biomarkers. The metabolites were ranked according to the area under the ROC curve (AUC) values in heat maps, which were used to illustrate the discriminatory power of potential biomarkers (Fig. 4A). All AUC values were between 0.663 and 0.941. Because the ESCC is a complex disease that involves the systemic disorder of biochemical pathways, a biomarker panel containing a group of biomarkers rather than a single biomarker could be more powerful to discriminate and provide pathophysiology information. Therefore, metabolites with AUC > 0.85 were analyzed by binary logistic regression combined with ROC curves to build the biomarker panel. The results (Fig. 4B) showed that the panel of five metabolites (decanoylcarnitine, cAMP, heptanoylcarnitine, cGMP, and phenylacetylglutamine) had an AUC of 0.981. The values of sensitivity and specificity reached 91.3% and 98.4%, respectively, at the best cut-off points. These results indicated that the biomarker panel could provide more reliable discrimination between ESCC patients and normal controls. In the future, larger urine samples will be acquired to validate these conclusions.

### Biological significance of biomarkers

**Figure 5:**
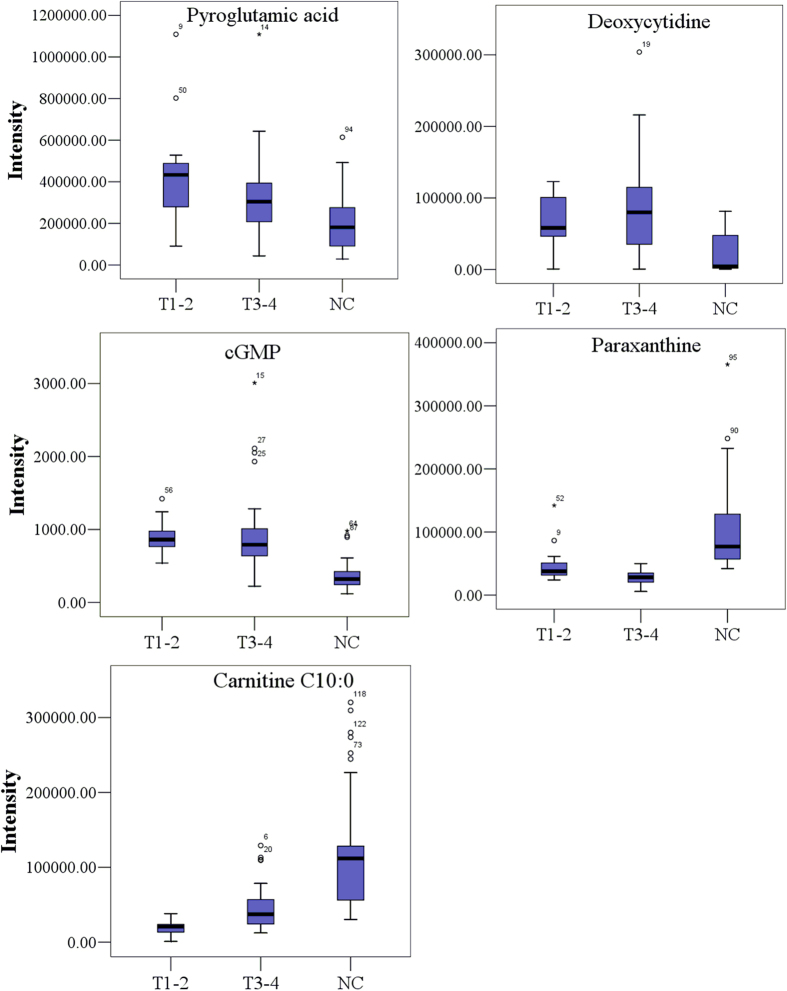
Typical metabolite variations in urine samples from T1–2 and T3–4 ESCC patients relative to controls.

Among the 19 identified potential biomarkers, 11 were up-regulated, and eight down-regulated in patients (Table 2). The trends in the levels of these potential biomarkers in T1–2 and T3–4 ESCC patients relative to controls were further analyzed, and representative metabolites are shown in Fig. 5.

## Discussion

EC is an aggressive malignancy with poor prognosis due to the delayed diagnosis in part. Metabolomics, which is developed in recent years, offers a novel, convenient and sensitive approach to get the disturbed metabolic pathways and the turmor-associated biomarkers. Several metabolomics studies of EC have been carried out. Using NMR-based approach, Davis, V. W. *et al*. performed the urinary metabolomics of EC. The results showed clear distinctions between EC, Barrett’s esophagus and healthy controls, and the related biomarkers were discovered. However, the patients in this study were limited to esophageal adenocarcinoma (EAC)^23^. Jin, H. *et al*. performed the serum metabolomics signatures of lymph node metastasis of ESCC with gas chromatography (GC)-MS. A series of differential metabolites in serum for ESCC and lymph node metastatic ESCC patients were discovered and identified, and finally a potential biomarker panel (valine, γ-aminobutyric acid and pyrrole-2-carboxylic acid) were screened for ESCC diagnosis^25^. Previously, we also carried out the LC-MS-based plasma metabolomics of ESCC, and some potential biomarkers were discovered for diagnosis and therapeutic effect prediction^27^. In present study, the ESCC urinary metabolomics based on LC-MS approach was performed to discover the biomarkers for disease diagnosis and staging. The results might offer the supplement information for the previous studies, and be useful for the ESCC diagnosis.

Carnitine and acylcarnitines, which are intermediates in the key energy metabolic pathways of fatty acid β-oxidation, are present at different concentrations in the urine of ESCC patients than in the urine of matched control patients (Table 2). Carnitines play an important role in transporting long-chain fatty acids across the mitochondrial membranes and short-chain fatty acids across mitochondria into the cytosol, and further participate in β-oxidation and energy metabolism^34^,^35^. The present results indicated that fatty acid β-oxidation was disturbed in ESCC patients. This finding is consistent with the Warburg effect, in which most cancer cells preferentially utilize glycolysis over other forms of energy production, including fatty-acid oxidation through acetyl-CoA^36^,^37^. The carnitine system in cancers has also been explored in previous studies examining other diseases^38^,^39^. Our previous results based on the ESCC plasma metabolomics also revealed that the levels of carnitines changed in patients^27^. This study provides the complementary information about the relationship between carnitines and cancers, especially for ESCC. The results may establish not only a new screening method, but also identify a new therapeutic target for this disease. For example, promoters of fatty acid β-oxidation might be evaluated to determine whether they have salutary effects on ESCC cells *in vitro*.

The dramatic increases in the levels of phenylacetylglutamine, pyroglutamic acid, urocanic acid, and indoxyl indicate that amino acid metabolism is disturbed in ESCC patients. Phenylacetylglutamine, a normal constituent of human urine, forms in the liver following the condensation of glutamine with phenylacetyl-CoA^40^. The urinary levels of phenylacetylglutamine have been used to monitor surrogate liver glutamate and to investigate a liver citric acid cycle intermediate^41^. Pyroglutamic acid, a cyclized derivative of L-glutamic acid, is formed nonenzymatically from glutamate, glutamine, and γ-glutamylated peptides, but it can also be produced through the action of γ-glutamylcyclotransferase on L-amino acid^42^. Elevated urine levels of pyroglutamic acid may be associated with glutamine metabolism problems. Urocanic acid is an intermediate in the conversion of histidine to glutamic acid. The up-regulation of urinary urocanic acid could result from a histidine metabolism disorder in tumor tissue and/or a glutamic acid metabolism disorder. Indoxyl is reported with tryptophan metabolism in kyoto encyclopedia of genes and genomes (kegg) pathway analysis. The hydrolysis of tryptophan yields indole, and the oxidation of indole produces indoxyl.

Purine and pyrimidine metabolism were also abnormal in ESCC patients relative to controls. The cAMP and cGMP levels were significantly altered in ESCC patients. cAMP, the first second messenger to be identified, plays fundamental roles in cellular responses to many hormones and neurotransmitters^43^. cGMP, a ubiquitous second messenger, mediates several signal transduction pathways in mammalian cells^44^. Increasing evidence suggests that cGMP plays an important role in cellular proliferation, differentiation, and apoptosis^45^. Uric acid, produced by the enzyme xanthine oxidase during purine metabolism^46^, was found to be up-regulated in ESCC patients’ urine samples, as observed in plasma^27^. Paraxanthine, the preferential product of caffeine metabolism in humans, is formed by the demethylation of caffeine by P450 1A2 in the liver. The reduction of paraxanthine may indicate that the metabolic activity of P450 1A2 declined^47^. Deoxycytidine is the intermediate or end-product of nucleotide and nucleic acid metabolism^48^. The increase in deoxycytidine might stem from abnormal cell proliferation in cancer tissue.

Cancer is considered to be a complex disease involving the systemic deregulation of cell proliferation, survival, apoptosis, and the cell cycle. Consequently, it can lead to disorders of some related metabolic pathways. Therefore, a biomarker panel might be more effective than a single biomarker for diagnosing cancer patients and elucidating the pathophysiology of cancer. It should be noted that the biomarker panel described here may not be unique to ESCC. Further validation of a highly ESCC-specific biomarker panel, including larger cohorts of different patients, will be conducted in the near future.

## Conclusions

Identifying metabolic biomarkers can contribute to improving diagnostics, prognostication, and therapy. Because the development and prognosis of ESCC varies significantly with genetic background, noninvasive ESCC biomarkers would significantly improve screening and diagnosis. Urinary metabolomics offers a novel and sensitive approach to simultaneously evaluating tumor-associated perturbations of multiple metabolic pathways and their downstream functional significance prior to the appearance of gross phenotypic changes.

In this study, we coupled LC-MS with MVDA to perform global urine metabolomics analysis of ESCC. The resulting data clearly demonstrated differences between patients and healthy controls. Furthermore, the data from ESCC patients clustered according to the cancer stage. Finally, we identified 19 metabolites as potential diagnostic biomarkers and studied their related metabolic pathways. Significant differences in these biomarkers suggest that ESCC patients have disorders in fatty acid β-oxidation; amino acid, purine, pyrimidine metabolism; and fructose or mannose degradation. Importantly, metabolites are regulated by both intrinsic and extrinsic factors, and thus, the specificity of these endogenous markers must be further evaluated. Further studies will be conducted to validate these biomarkers in larger cohorts of different patients. This study confirmed the feasibility of using a LC-MS-based urine metabolomics platform to characterize ESCC.

## Methods

### Chemicals

High-performance LC (HPLC)-grade acetonitrile (ACN) and formic acid (FA) were obtained from Merck (Germany). Standard compounds, including L-phenylalanine, hippuric acid, hydrocortisone, estrone, tryptophan, cholic acid, linoleic acid, urocanic acid, L-fucose, L-carnitine, uric acid, deoxycytidine, cAMP, and cGMP were purchased from Sigma-Aldrich (USA). Phenylacetylglutamine, trioxymethylanthraquinone, and 2-hydroxybenzoic acid were purchased from the National Institute for the Control of Pharmaceutical and Biological Products (China).

### Sample Collection

Sixty-two ESCC patients and 62 healthy volunteers from the Cancer Institute and Hospital of the Chinese Academy of Medical Sciences (Beijing, China) were enrolled in the study. All patients were diagnosed by histopathological examination. No patients had received chemotherapy or radiation, and they had not undergone surgical operation before sample collection. ESCC stage was built due to the 2009 Tumor Node Metastasis (TNM) staging system. The detailed demographic profiles of the participants are provided in Table 3. The study was approved by the Cancer Institute and Hospital of the Chinese Academy of Medical Sciences ethics committee and with the approval of corresponding regulatory agencies, and all the experiments were carried out in accordance with the approved guidelines. Meanwhile, all the patients involved in the study signed the informed consent form and agreed to participate. All urine samples were collected before breakfast following the informed consent guidelines and immediately stored at −80 °C prior to sample preparation and analysis.

### Sample preparation

The urine samples were thawed at 4 °C before analysis. Creatinine analysis was performed by the Inspection Department of the Cancer Institute and Hospital of the Chinese Academy of Medical Sciences using an enzymatic procedure. The samples were prepared by centrifugation at 10,000 relative centrifugal force (rcf) at 4 °C for 5 min, followed by creatinine value-calibrated dilution. A pooled QC) sample was prepared by mixing the same volume (10 μL) of each sample^49^. Moreover, a mixed standard including (1) L-carnitine, (2) L-phenylalanine, (3) hippuric acid, (4) hydrocortisone, (5) estrone, (6) tryptophan, (7) cholic acid, and (8) linoleic acid was also used to monitor the stability of the analytical system.

### LC-MS analysis

The study was performed on a 1200 Series Rapid Resolution Liquid Chromatography system (Agilent Technologies, Germany) coupled to a quadrupole time-of-flight (Q-TOF) mass spectrometer (QSTAR Elite, AB Sciex, USA) equipped with ESI sources. The system was controlled by Analyst QS 2.0 (QSTAR Elite, AB Sciex, USA).

A 10 cm × 2.1 mm Zorbax Aq-C_18_ 1.8 μm column was used and maintained at 60 °C. The mobile phase was (A) 0.1% FA-water and (B) ACN, with multi-step gradient conditions as follows: initial 0% B maintained for 8 min, then increased to 10% B in 5 min; increased to 60% B over 5–15 min; 15–20 min to 100% B, and finally maintained at 100% B for 8 min, at a flow rate of 200 μL/min. The injection volume was 5 μL for each run. Healthy volunteers and ESCC patients samples were analyzed in random order. QC and mixed-standard samples were also analyzed repeatedly within the analytical run after every ten plasma samples to evaluate chromatographic reproducibility.

The LC-MS data were acquired in both positive and negative ion modes. The detailed parameters were as follows: spray voltage 5.5 kV or −4.5 kV, declustering voltage 50 V or −50 V, vaporizer temperature 375 °C, turbo gas 75 psi, nebulizer gas 65 psi, curtain gas 40 psi. Full Scan analysis was performed in TOF mode with the scan range of *m/z* 65–850, and the MS/MS analysis was accomplished with information-dependent acquisition (IDA) mode with collision energy (CE) 40/20 or −35/−20 eV. Acquired data were auto-calibrated by background ions in positive ion mode (phthalates: *m/z* 149.0233 and *m/z* 391.2843) and standard solutions (2-hydroxybenzoic acid: *m/z* 137.0244 and trioxymethylanthraquinon *m/z* 269.0455) which were introduced by post-column mixing in negative ion mode.

### Data preprocessing and MVDA analysis

Freely available software XCMS version 2.10.0 and commercial software SIMCA-P version 12.0 (Umetrics AB, Umeå, Sweden)were used in this study. Raw data obtained by LC-MS analysis was firstly converted to the mzData format by the Wiff-to-mzData translator and then imported into XCMS software^50^ (http://masspec.scripps.edu/xcms/xcms.phpUT). Parameters for detailed data preprocessing in XCMS are available in the Supporting Information. After the data preprocessing, the SIMCA-P was further adopted for MVDA of the resultant 2D data matrices with mean centering and pareto scaling. Principal component analysis (PCA) was used to visualize the stability of the system. The cross-validation was used to test the model validity against overfitting. Potential biomarker candidates were selected based on variable importance in project (VIP > 1), S-plot, and the raw data plot in orthogonal partial least-square discriminant analysis (OPLS-DA) model, and independent t-test (*P* < 0.05). Finally, the fragment, isotope and adduct ions were manually removed according to the corresponding extracted ion chromatograms (XICs) and the potential biomarkers were screened out.

### Metabolite identification and characterization

The structure of potential biomarkers was identified as described^27^,^33^,^51^,^52^, firstly by searching the free databases such as HMDB (http://hmdb.ca), Massbank (http://massbank.imm.ac.cn/MassBank), and METLIN (http://metlin.scripps.edu) with exact molecular weights; and then using high-resolution LC-MS/MS spectra for further identification; applying standard compounds to verify the potential structures; and finally obtained the identification score by the scoring metric.

The discriminatory power of potential biomarkers was characterized by the area under the ROC curve (AUC) produced by SPSS (version 17.0)^53^, and visually displayed by heat maps^54^.

## Additional Information

**How to cite this article**: Xu, J. *et al*. Global metabolomics reveals potential urinary biomarkers of esophageal squamous cell carcinoma for diagnosis and staging. *Sci. Rep.* **6**, 35010; doi: 10.1038/srep35010 (2016).

## Supplementary Material

Supplementary Information

## Figures and Tables

**Table 1:** Retention times of mixed standards peaks detected by LC-MS.

Compounds	LC-(+)ESI-MS			
	Rt (min)	RSD (%)	Peak Area (×10^5^)	RSD (%)
L-Carnitine	1.4	0.86	9.788	5.01
Phenylalanine	2.9	0.16	6.415	2.44
Hippuric acid	8.2	1.50	3.170	2.56
Hydrocortisone	15.0	0.51	18.375	4.20
Estrone	17.4	0.2	0.317	11.14
**LC-(−)ESI-MS**				
Phenylalanine	2.9	1.22	1.569	4.33
Hippuric acid	7.5	2.56	5.916	3.47
Tryptophan	5.1	0.86	1.674	4.02
Cholic acid	13.5	0.52	1.406	10.35
Linoleic acid	20.9	0.38	8.785	5.26

**Table 2:** Urine potential biomarkers associated with ESCC.

No.	RT(min)	*m/z*	metabolite identification	*P*-value^c^	VIP^d^	trend^e^	related pathway	The identification score^f^
1	9.43	130.0501	Pyroglutamic acid^b^	3.65E-06	6.59	↑	Glutamine metabolism	6
2	7.45	134.0591	Indoxyl^b^	0.006	1.52	↑	Tryptophan metabolism	6
3	2.27	139.0507	Urocanic acid^a,b^	0.017	1.53	↑	Histidine metabolism	8
4	1.45	162.1097	L-Carnitine^a,b^	0.021	2.79	↑	Fatty acid transportation	8
5	2.27	165.0554	L-Fucose^a,b^	0.003	1.88	↑	Fructose and mannose degradation	8
6	2.89	169.0378	Uric acid^a,b^	4.89E-05	7.12	↑	Purine metabolism	8
7	9.56	181.0719	Paraxanthine^b^	0.012	2.31	↓	Caffeine metabolism	6
8	2.07	204.1249	Acetylcarnitine^b^	0.025	6.65	↑	Fatty acid β oxidation	6
9	3.41	228.0801	Deoxycytidine^a,b^	2.51E-09	4.36	↑	Pyrimidine metabolism	8
10	9.43	265.1169	Phenylacetylglutamine^a,b^	7.8E-07	12.91	↑	Phenylalanine metabolism	8
11	11.93	274.2009	Heptanoylcarnitine (carnitine C 7:0)^b^	0.031	1.69	↓	Fatty acid β oxidation	6
12	12.3	286.2005	Octenoylcarnitine (carnitine C 8:1)	0.008	2.13	↓	Fatty acid β oxidation	4
13	13.39	300.2163	Nonenoylcarnitine (carnitine C 9:1)	0.025	1.39	↓	Fatty acid β oxidation	4
14	14.03	302.2319	Nonanoylcarnitine (carnitine C 9:0)^b^	0.0025	3.39	↓	Fatty acid β oxidation	6
15	14.92	316.2472	Decanoylcarnitine (carnitine C 10:0)^a,b^	0.012	1.36	↓	Fatty acid β oxidation	8
16	15.14	328.2473	Undecenoylcarnitine (carnitine C 11:1)	0.019	1.78	↓	Fatty acid β oxidation	4
17	5.08	330.0588	cAMP^a,b^	0.016	2.15	↑	Purine metabolism	8
18	11.70	330.2650	Undecanoylcarnitine (carnitine C 11:0)^b^	0.019	1.45	↓	Fatty acid β oxidation	6
19	4.94	346.0547	cGMP^a,b^	2E-14	3.17	↑	Purine metabolism	8

^a^Metabolites confirmed by standard compounds. ^b^Metabolites provisionally identified by database searches and MS fragmentation. Others, proposals based on MS fragmentation and exact mass data. ^c^*P* value of independent *t*-test. ^d^VIP is variable importance in the projection obtained from OPLS-DA with a threshold of 1.0. ^e^Change trend compared with controls. (↑): up-regulated. (↓): down-regulated. ^f^The identification score is calculated by the scoring metric.

**Table 3:** Clinicopathologic characteristics of the study samples.

characteristics	ESCC patients	Healthy controls
No. of subjects	62	62
Age (mean, range)	62, 46–78	60, 45–74
BMI (mean, range)	22.1, 16.4–30.4	21.6, 16.7–29.6
Gender	male	male
Race	Chinese	Chinese
Cancer stage		
Early stage (T1–2, without metastases)	22 (T1: 7, T2: 15)	**—**
Advanced stage (T3–4, with metastases)	40 (T3: 19, T4: 21)	**—**
