# Supplementary material for: Global metabolomics reveals potential urinary biomarkers of esophageal squamous cell carcinoma for diagnosis and staging
Source: Sci Rep. 2016 Oct 11;6:35010. doi: 10.1038/srep35010 (PMC5057114; doi:10.1038/srep35010)

**Supporting Information**

Global metabolomics reveals potential urinary biomarkers of esophageal squamous cell carcinoma for diagnosis and staging

Jing Xu^1^, Yanhua Chen^1^, Ruiping Zhang^1^, Jiuming He^1^, Yongmei Song^2^, Jingbo Wang^3^, Huiqing Wang^1^, Luhua Wang^3^, Qimin Zhan^2^, Zeper Abliz^1, 4^*

^1^ State Key Laboratory of Bioactive Substance and Function of Natural Medicines, Institute of Materia Medica, Chinese Academy of Medical Sciences & Peking Union Medical College, Beijing 100050, P. R. China

^2^ State Key Laboratory of Molecular Oncology, Cancer Institute & Hospital, Chinese Academy of Medical Sciences & Peking Union Medical College, Beijing 100021, P. R. China

^3^ Department of Radiation Oncology, Cancer Institute & Hospital, Chinese Academy of Medical Sciences & Peking Union Medical College, Beijing 100021, P. R. China

^4^ Centre for Bioimaging & Systems Biology, Minzu university of China, Beijing 100081,P. R.

China

Corresponding author. Prof. Zeper Abliz

Tel: 86-10-63165218;

Fax: 86-10-63165218

E-mail: [zeper@imm.ac.cn](mailto:zeper@imm.ac.cn)

**The program used for peak discrimination, filtering, alignment and CAMERA analysis in**

**LC-(+)ESI-MS data.**

rm(list=ls(all=TRUE))

library(Biobase)

library(xcms)

library(multtest)

library(CAMERA)

sessionInfo()

xs<-xcmsSet(profmethod="binlin",method="centWave",ppm=60,peakwidth=c(8,25),prefilter=c(3,60),snthresh =20, mzdiff =0.05)

xs

xs <-group(xs,bw=10,minfrac=0.5)

save(xs,file="xs.Rda")

ret.xs.obiwarp <-retcor(xs,method="obiwarp",plottype="deviation")

ret.xs.obiwarp<-group(ret.xs.obiwarp, bw = 10)

ret.xs.obiwarp

fill.ret.xs.obiwarp<-fillPeaks(ret.xs.obiwarp)

fill.ret.xs.obiwarp

save(fill.ret.xs.obiwarp, file="fill.ret.xs.obiwarp.Rda")

an.ESCC<-annotate(fill.ret.xs.obiwarp,sigma=6,perfwhm=0.3,cor_eic_th=0.75,maxcharge=3,maxiso=3,mzabs=0.03,multiplier=3,polarity="positive",category="ESCC")

peaklist.ESCC<-getPeaklist(an.ESCC)

write.csv(peaklist.ESCC,file='annotated.ESCC.csv')

an.NC<-annotate(fill.ret.xs.obiwarp,sigma=6,perfwhm=0.3,cor_eic_th=0.75,maxcharge=3,maxiso=3,mzabs=0.03,multiplier=3,polarity="positive",category="NC")

peaklist.NC<-getPeaklist(an.NC)

write.csv(peaklist.NC,file='annotated.NC.csv')

report.fill.ret.xs.obiwarp<-diffreport(fill.ret.xs.obiwarp,"ESCC","NC",eicmax=5000,file=" c18-pos")

save(report.fill.ret.xs.obiwarp, file="report.fill.ret.xs.obiwarp.Rda")

**The program used for peak discrimination, filtering, alignment and CAMERA analysis in**

**LC-(-)ESI-MS**

rm(list=ls(all=TRUE))

library(Biobase)

library(xcms)

library(multtest)

library(CAMERA)

sessionInfo()

xs<-xcmsSet(profmethod="binlin",method="centWave",ppm=60,peakwidth=c(8,25),snthresh=20,prefilter=c(3,60), mzdiff =0.05)

xs

xs <-group(xs,bw=10,minfrac=0.5)

save(xs,file="xs.Rda")

ret.xs.obiwarp <-retcor(xs,method="obiwarp",plottype="deviation")

ret.xs.obiwarp<-group(ret.xs.obiwarp, bw=10,minfrac=0.5)

ret.xs.obiwarp

save(ret.xs.obiwarp, file="ret.xs.obiwarp.Rda")

fill.ret.xs.obiwarp<-fillPeaks(ret.xs.obiwarp)

fill.ret.xs.obiwarp

save(fill.ret.xs.obiwarp, file="fill.ret.xs.obiwarp.Rda"

an.ESCC<-annotate(fill.ret.xs.obiwarp,sigma=6,perfwhm=0.3,cor_eic_th=0.75,maxcharge=3,maxiso=3,mzabs=0.03,multiplier=3,polarity="negative",category="ESCC")

peaklist.ESCC<-getPeaklist(an.ESCC)

write.csv(peaklist.ESCC,file='annotated.ESCC.csv')

an.NC<-annotate(fill.ret.xs.obiwarp,sigma=6,perfwhm=0.3,cor_eic_th=0.75,maxcharge=3,maxiso=3,mzabs=0.03,multiplier=3,polarity="negative",category="NC")

peaklist.NC<-getPeaklist(an.NC)

write.csv(peaklist.NC,file='annotated.NC.csv')

report.fill.ret.xs.obiwarp<-diffreport(fill.ret.xs.obiwarp,"ESCC","NC",eicmax=5000,file=" c18-neg")

save(report.fill.ret.xs.obiwarp, file="report.fill.ret.xs.obiwarp.Rda")

**Figure legends**

**Figure S1.** Flowchart of the LC-MS-based urine metabolomics approach to ESCC diagnosis and staging。

**Figure S2.** PCA score plots of all samples (including ESCC, NC and QC) from (A) LC-(+)ESI-MS and (B) LC-(-)ESI-MS. ●ESCC and NC; ■ QC

**Figure S3**. The XICs of mix standard with LC-ESI-MS analysis on a Q-TOF instrument in (A) positive ion mode and (B) negative ion mode. (1)L-carnitine, (2)L-phenylalanine, (3)hippuric acid, (4)hydrocortisone, (5)estrone, (6)tryptophan, (7)cholic acid, and (8)linoleic acid.

**Figure S4.** The retention time deviation profiles deriving from (A) LC-(+)ESI-MS and (B) LC-(-)ESI-MS analyses. A positive deviation indicates that the sample was eluting after the median retention time, and a negative deviation indicates that the samples was eluting before the median retention time.

**Figure S5**. The typical total ion chromatograms (TICs) of LC-MS in both positive and negative ion modes

**Figure S6.** PCA score plots based on the data from (A) LC-(+)ESI-MS and (B) LC-(-)ESI-MS of the ESCC patients and controls (●ESCC and ●NC).

**Figure S7.** PLS-DA validation plots of 999 random permutations between early stage ESCC patients (T1-2) and advanced stage ESCC patients (T3-4) based on the data from (A) LC-(+)ESI-MS and (B) LC-(-)ESI-MS.

**Figure S8.** MS data used for identification of Pyroglutamic acid. A: MS spectrum. B: MS/MS spectrum of *m/z* 130(DP=50 V, CE=20 eV).

**Figure S9.** MS data used for identification of Indoxyl. A: MS spectrum. B: MS/MS spectrum of *m/z* 134 (DP=50 V, CE=20 eV).

**Figure S10.** MS data used for identification of Urocanic acid. A: MS spectrum. B: MS/MS spectrum of *m/z* 139 (DP=50 V, CE=20 eV).

**Figure S11.** MS data used for identification of L-carnitine. A: A: MS spectrum. B: MS/MS spectrum of *m/z* 162.1 (DP=50 V, CE=20 eV).

**Figure S12.** MS data used for identification of L-Fucose. A: MS spectrum. B: MS/MS spectrum of *m/z* 165 (DP=50 V, CE=20 eV).

**Figure S13.** MS data used for identification of uric acid. A: MS spectrum. B: MS/MS spectrum of *m/z* 169 (DP=50 V, CE=20 eV).

**Figure S14.** MS data used for identification of paraxanthine. A: MS spectrum. B: MS/MS spectrum of *m/z* 181 (DP=50 V, CE=20 eV).

**Figure S15.** MS data used for identification of Acetylcarnitine. A: MS spectrum. B: MS/MS spectrum of *m/z* 204.1 (DP=50 V, CE=20 eV).

**Figure S16.** MS data used for identification of Phenylacetylglutamine. A: MS spectrum. B: MS/MS spectrum of *m/z* 265.1 (DP=50 V, CE=20 eV).

**Figure S17.** MS data used for identification of Heptanoylcarnitine. A: MS spectrum. B: MS/MS spectrum of *m/z* 274.2 (DP=50 V, CE=20 eV).

**Figure S18.** MS data used for identification of Octenoylcarnitine. A: MS spectrum. B: MS/MS spectrum of *m/z* 286.2 (DP=50 V, CE=20 eV).

**Figure S19.** MS data used for identification of Nonenoylcarnitine. A: MS spectrum. B: MS/MS spectrum of *m/z* 300.2 (DP=50 V, CE=20 eV).

**Figure S20.** MS data used for identification of Nonanoylcarnitine. A: MS spectrum. B: MS/MS spectrum of *m/z* 302.2 (DP=50 V, CE=20 eV).

**Figure S21.** MS data used for identification of Decanoylcarnitine. A: MS spectrum. B: MS/MS spectrum of *m/z* 316.2 (DP=50 V, CE=20 eV).

**Figure S22.** MS data used for identification of Undecenoylcarnitine. A: MS spectrum. B: MS/MS spectrum of *m/z* 328.2 (DP=50 V, CE=20 eV).

**Figure S23.** MS data used for identification of cAMP. A: MS spectrum. B: MS/MS spectrum of *m/z* 330 (DP=50 V, CE=20 eV).

**Figure S24.** MS data used for identification of Undecanoylcarnitine. A: MS spectrum. B: MS/MS spectrum of *m/z* 330.2 (DP=50 V, CE=20 eV).

**Figure S25.** MS data used for identification of cGMP. A: MS spectrum. B: MS/MS spectrum of *m/z* 346 (DP=50 V, CE=20 eV).

**Figure S1.** Flowchart of the LC-MS-based urine metabolomics approach to ESCC diagnosis and staging.


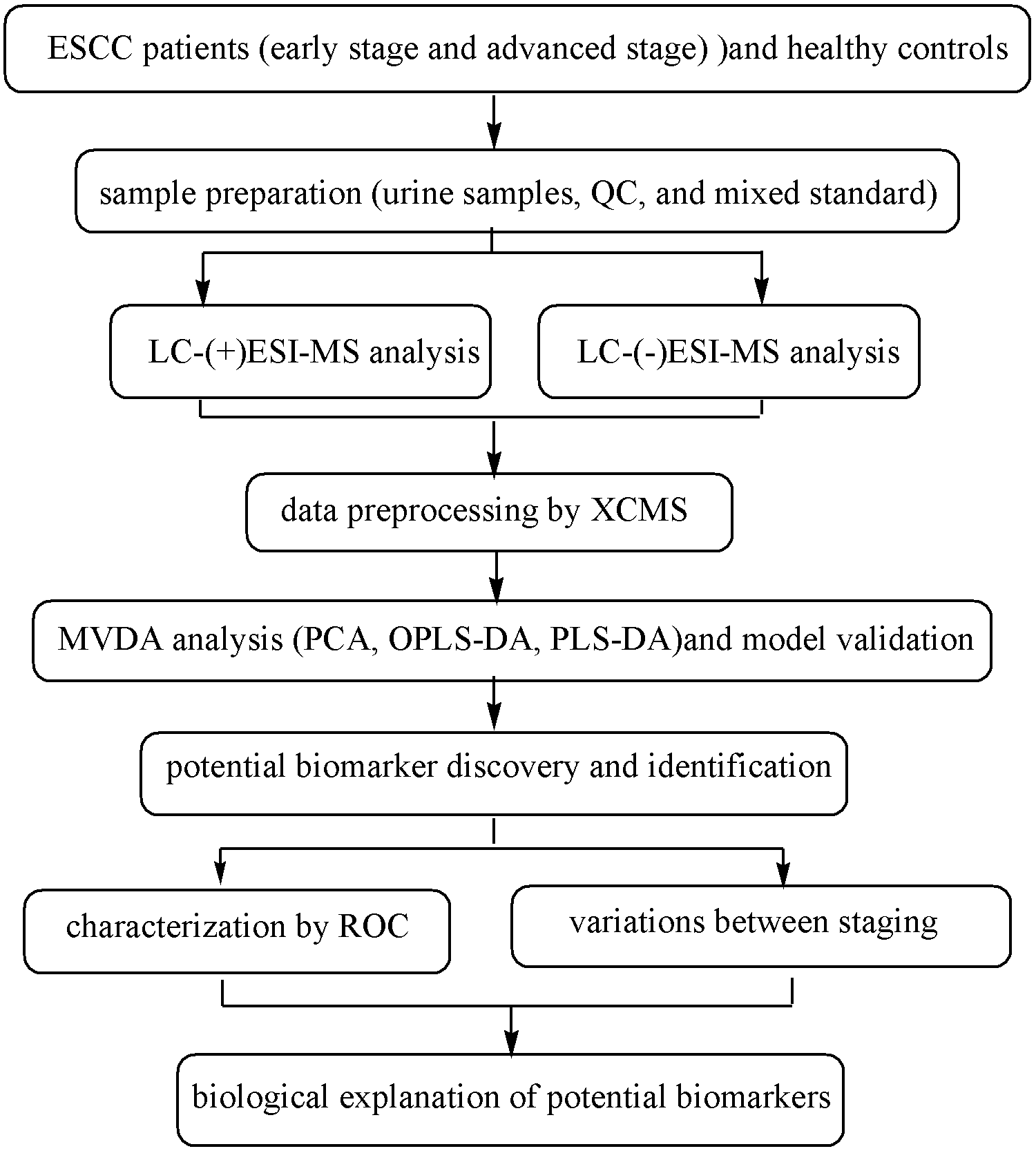


**Figure S2.** PCA score plots of all samples (including ESCC, NC and QC) from (A) LC-(+)ESI-MS and (B) LC-(-)ESI-MS. ●ESCC and NC; ■QC


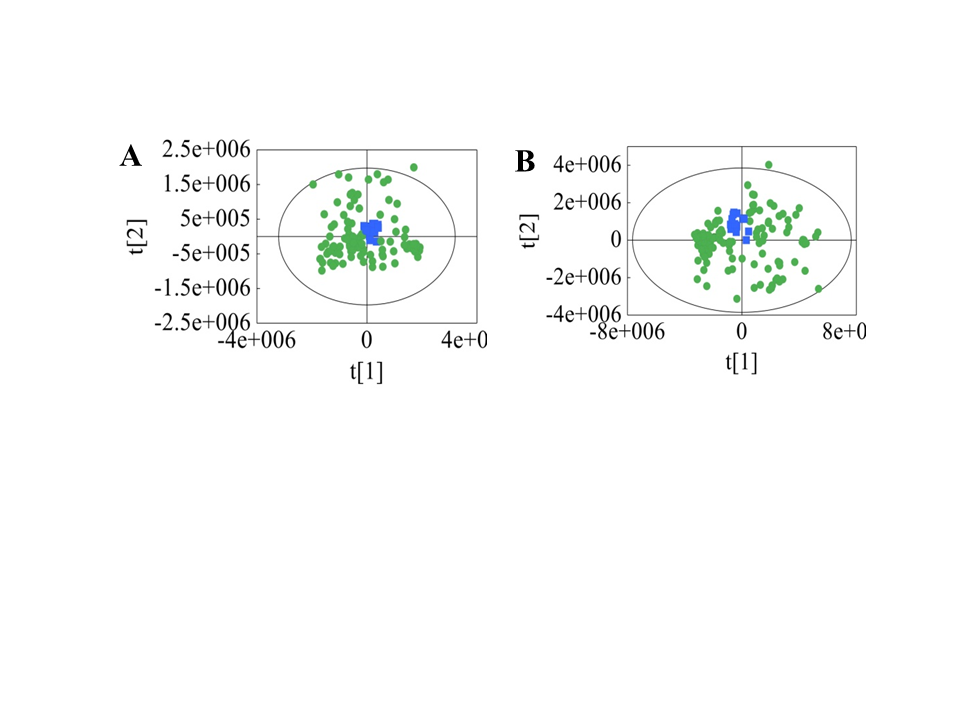


**Figure S3**. The XICs of mix standard with LC-ESI-MS analysis on a Q-TOF instrument in (A) positive ion mode and (B) negative ion mode. (1)L-carnitine, (2)L-phenylalanine, (3)hippuric acid, (4)hydrocortisone, (5)estrone, (6)tryptophan, (7)cholic acid, and (8)linoleic acid.

**Figure S4.** The retention time deviation profiles deriving from (A) LC-(+)ESI-MS and (B) LC-(-)ESI-MS analyses. A positive deviation indicates that the sample was eluting after the median retention time, and a negative deviation indicates that the samples was eluting before the median retention time.


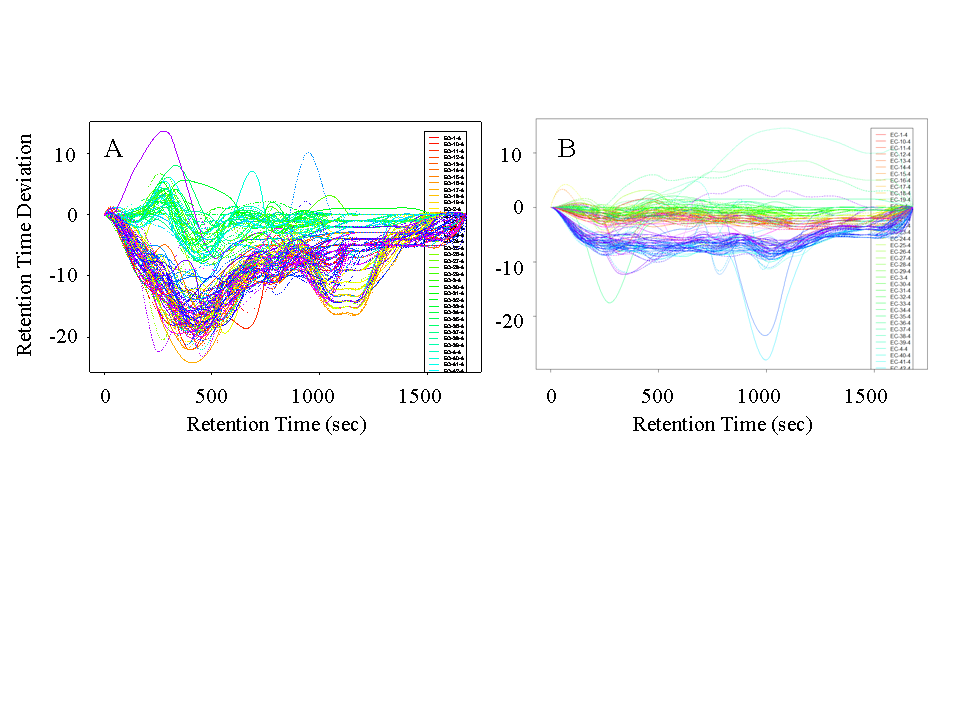


**Figure S5**. The typical total ion chromatograms (TICs) of LC- MS in both positive and negative ion modes

**Figure S6**. PCA score plots based on the data from (A) LC-(+)ESI-MS and (B) LC-(-)ESI-MS of the ESCC patients and controls (●ESCC and ●NC).

**Figure S7.** PLS-DA validation plots of 999 random permutations between early stage ESCC patients (T1-2) and advanced stage ESCC patients (T3-4) based on the data from (A) LC-(+)ESI-MS and (B) LC-(-)ESI-MS.


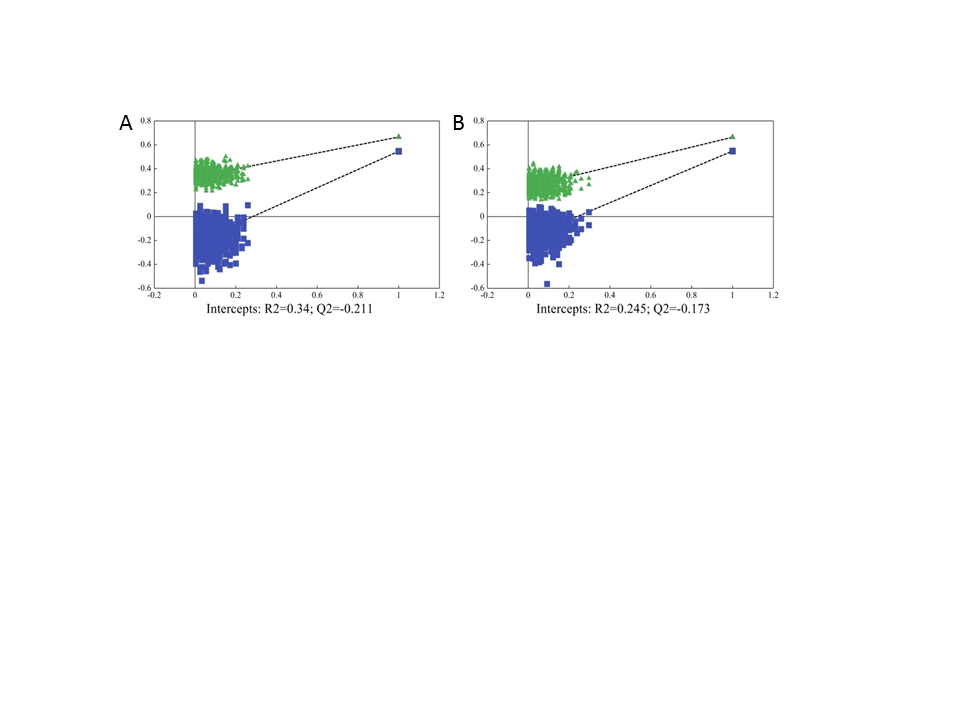


**Figure S8.** MS data used for identification of Pyroglutamic acid. A: MS spectrum. B: MS/MS spectrum of *m/z* 130 (DP=50 V, CE=20 eV).

**
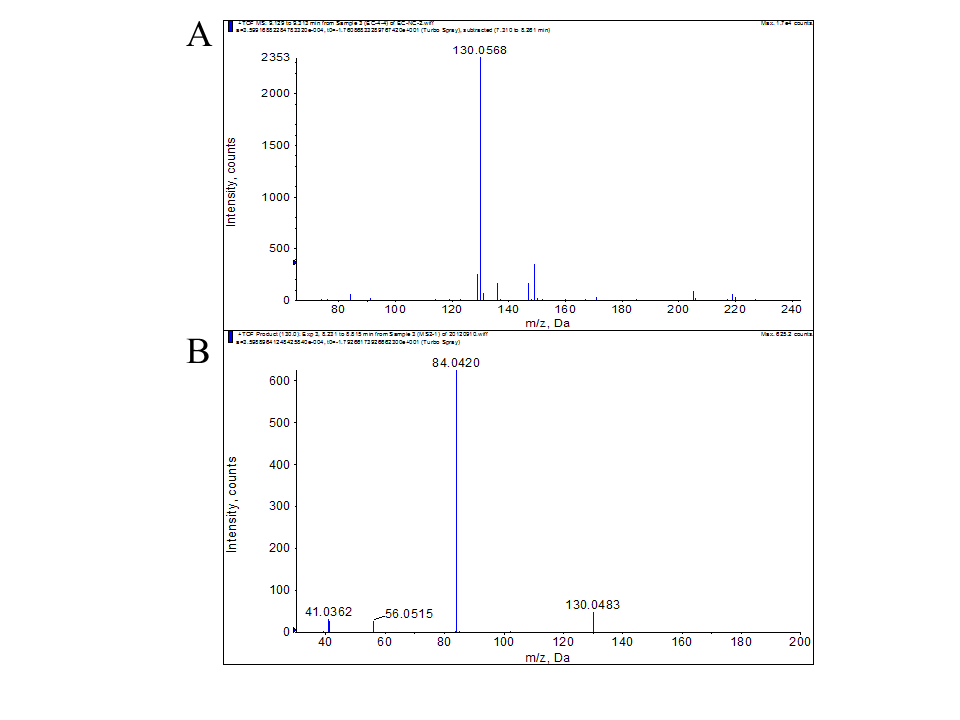
**

**Figure S9.** MS data used for identification of Indoxyl. A: MS spectrum. B: MS/MS spectrum of *m/z* 134 (DP=50 V, CE=20 eV).

**
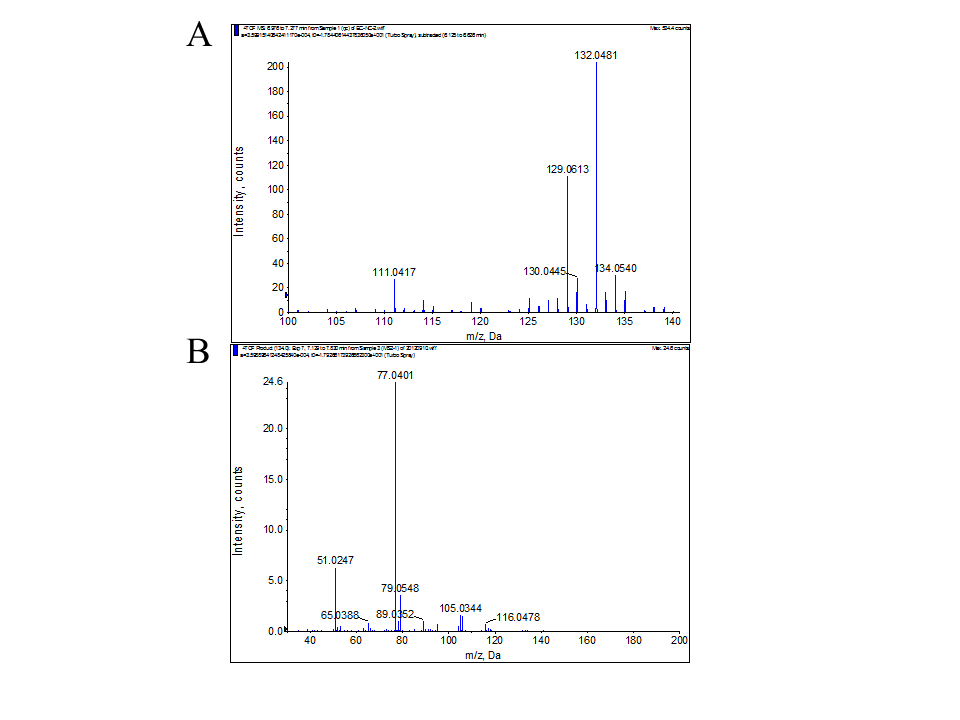
**

**Figure S10.** MS data used for identification of Urocanic acid. A: MS spectrum. B: MS/MS spectrum of *m/z* 139 (DP=50 V, CE=20 eV).

**
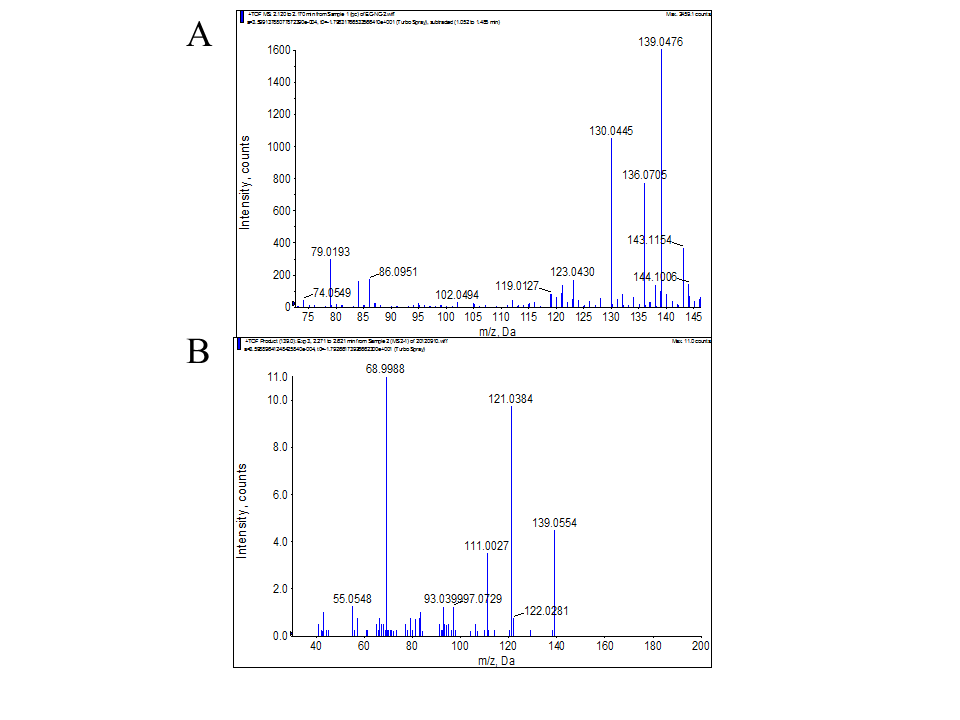
**

**Figure S11.** MS data used for identification of L-Carnitine. A: MS spectrum. B: MS/MS spectrum of *m/z* 162.1 (DP=50 V, CE=20 eV).

**
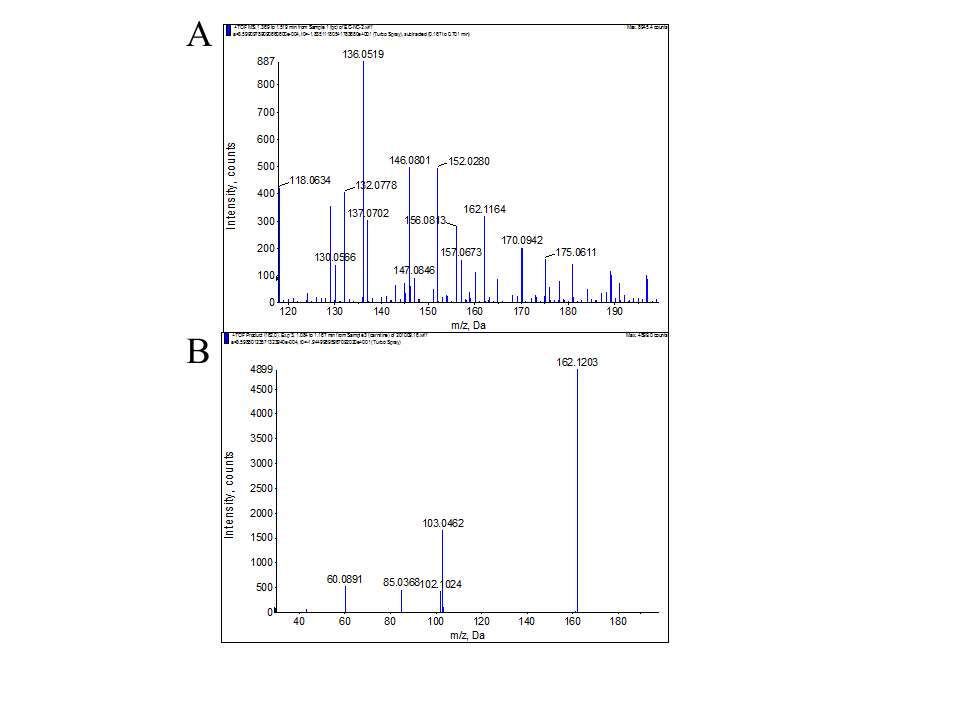
**

**Figure S12.** MS data used for identification of L-Fucose. A: MS spectrum. B: MS/MS spectrum of *m/z* 165 (DP=50 V, CE=20 eV).

**
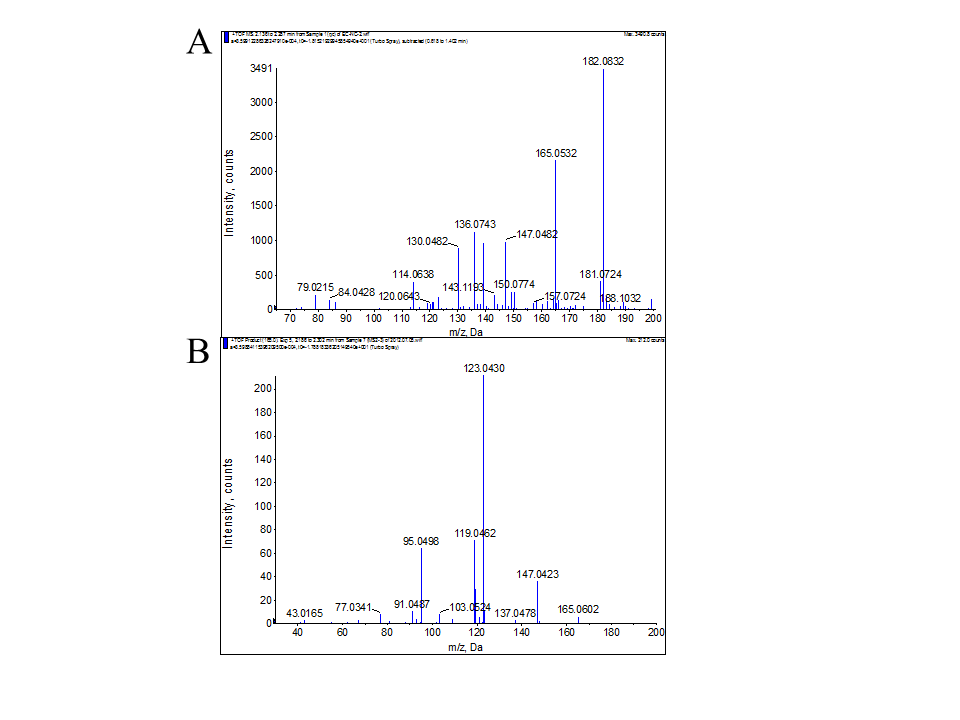
**

**Figure S13.** MS data used for identification of uric acid. A: MS spectrum. B: MS/MS spectrum of *m/z* 169 (DP=50 V, CE=20 eV).

**
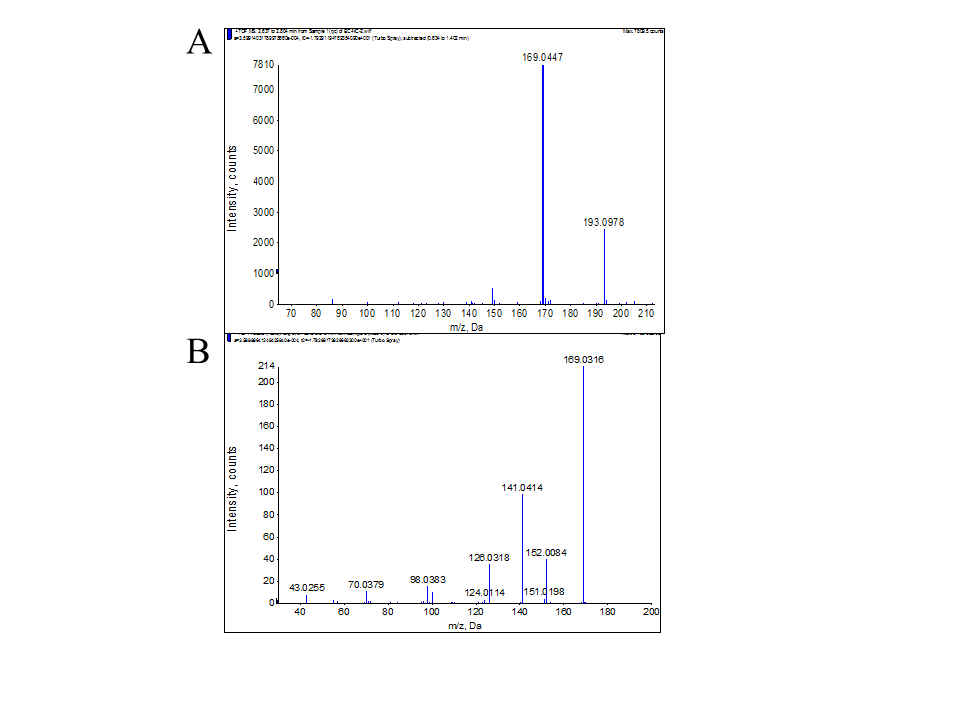
**

**Figure S14.** MS data used for identification of paraxanthine. A: MS spectrum. B: MS/MS spectrum of *m/z* 181 (DP=50 V, CE=20 eV).

**
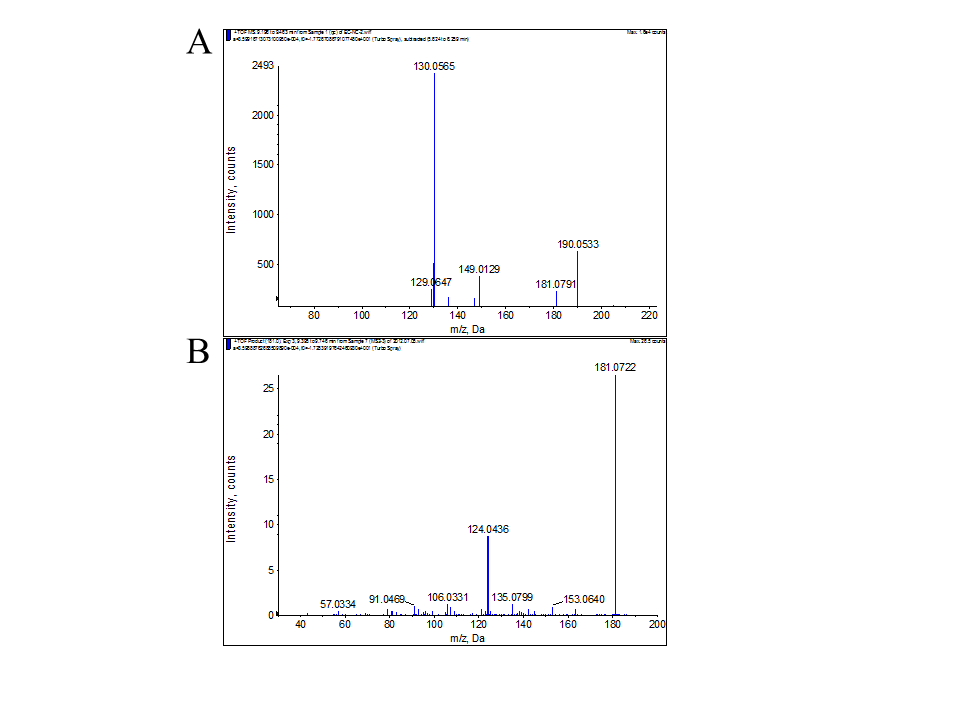
**

**Figure S15.** MS data used for identification of Acetylcarnitine. A: MS spectrum. B: MS/MS spectrum of *m/z* 204.1 (DP=50 V, CE=20 eV).

**
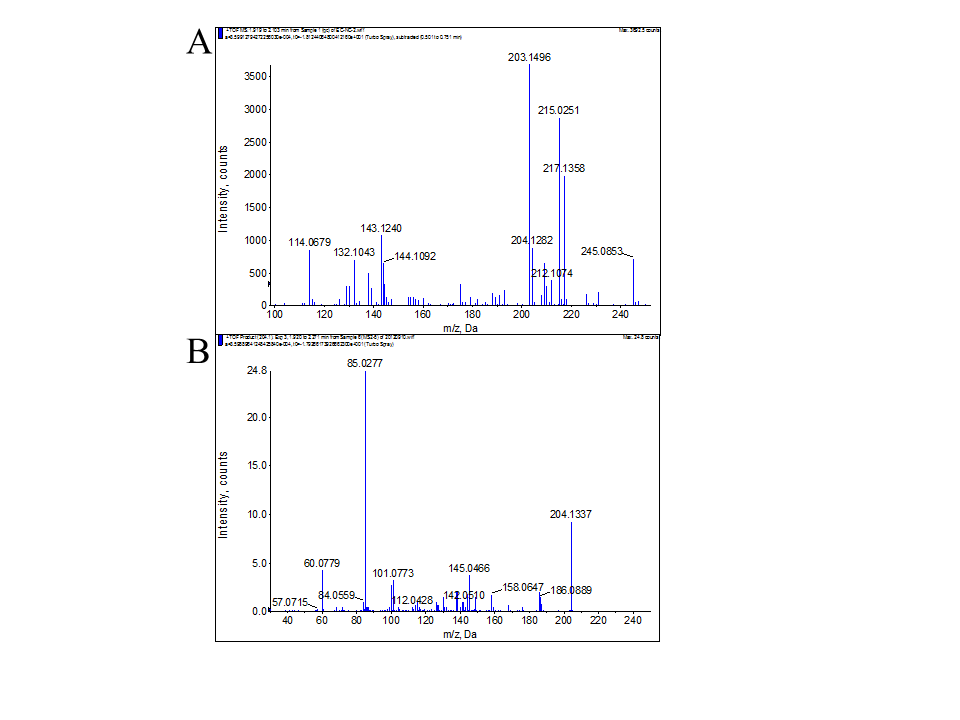
**

**Figure S16.** MS data used for identification of Phenylacetylglutamine. A: MS spectrum. B: MS/MS spectrum of *m/z* 265.1 (DP=50 V, CE=20 eV).


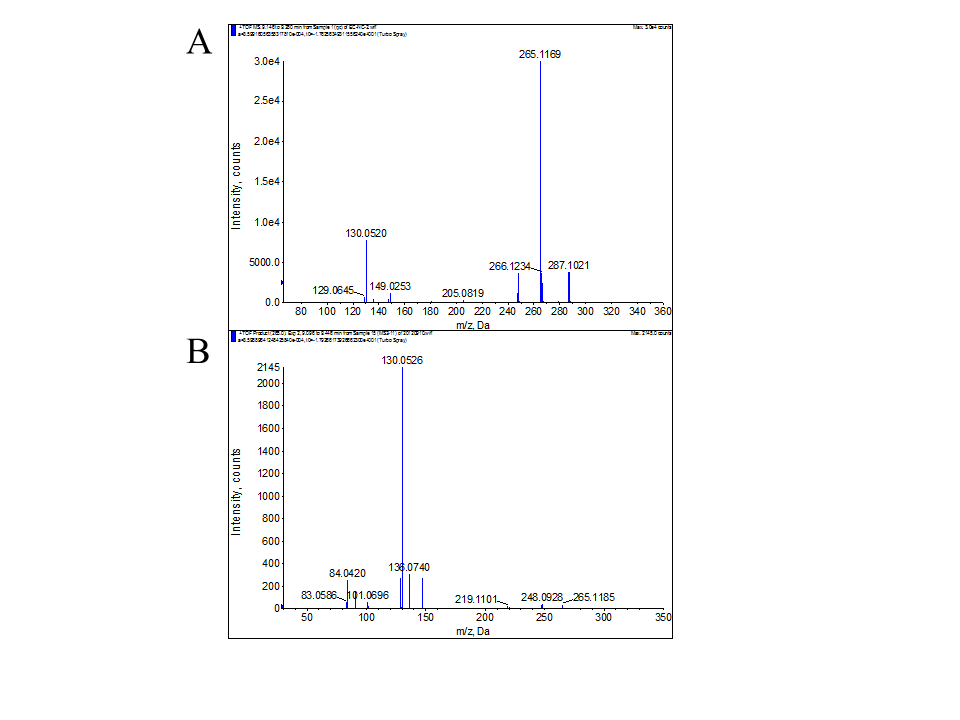


**Figure S17.** MS data used for identification of Heptanoylcarnitine. A: MS spectrum. B: MS/MS spectrum of *m/z* 274.2 (DP=50 V, CE=20 eV).


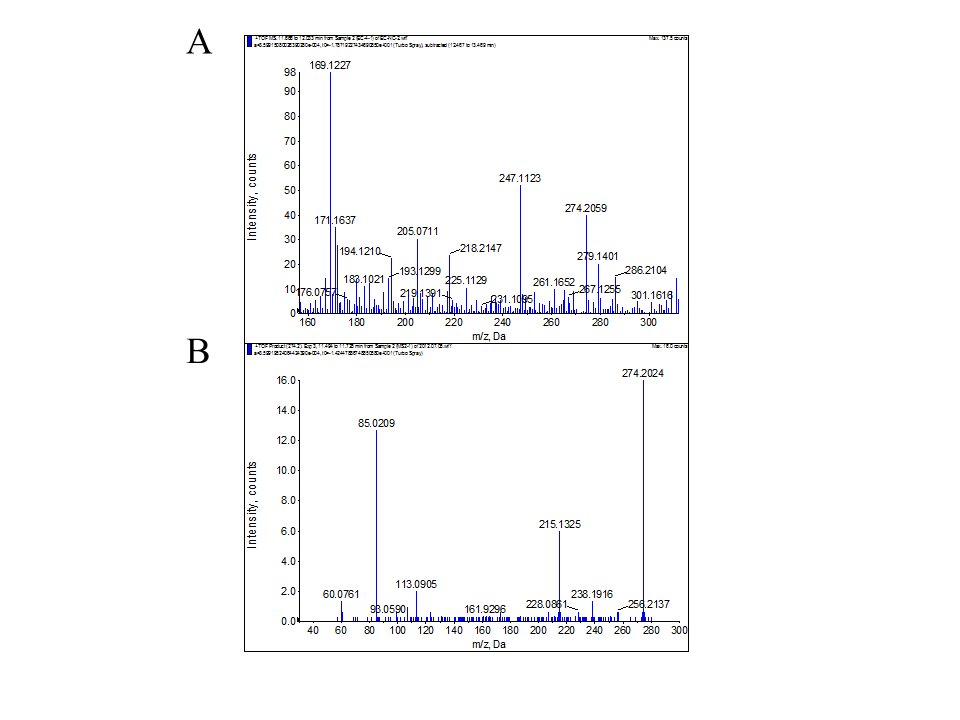


**Figure S18.** MS data used for identification of Octenoylcarnitine. A: MS spectrum. B: MS/MS spectrum of *m/z* 286.2 (DP=50 V, CE=20 eV).


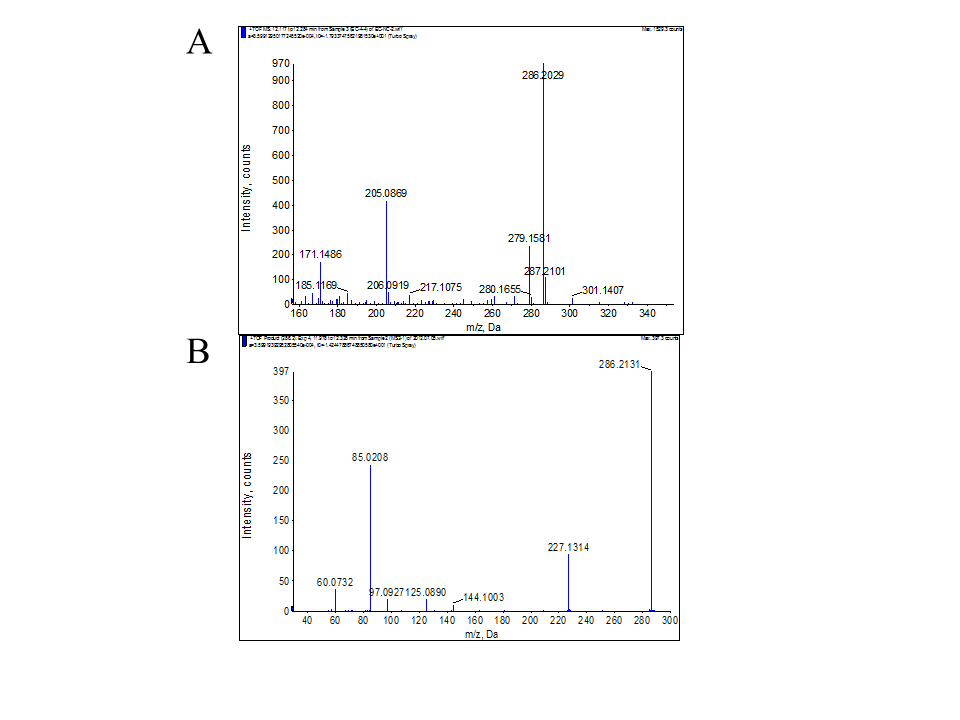


**Figure S19.** MS data used for identification of Nonenoylcarnitine. A: MS spectrum. B: MS/MS spectrum of *m/z* 300.2 (DP=50 V, CE=20 eV).


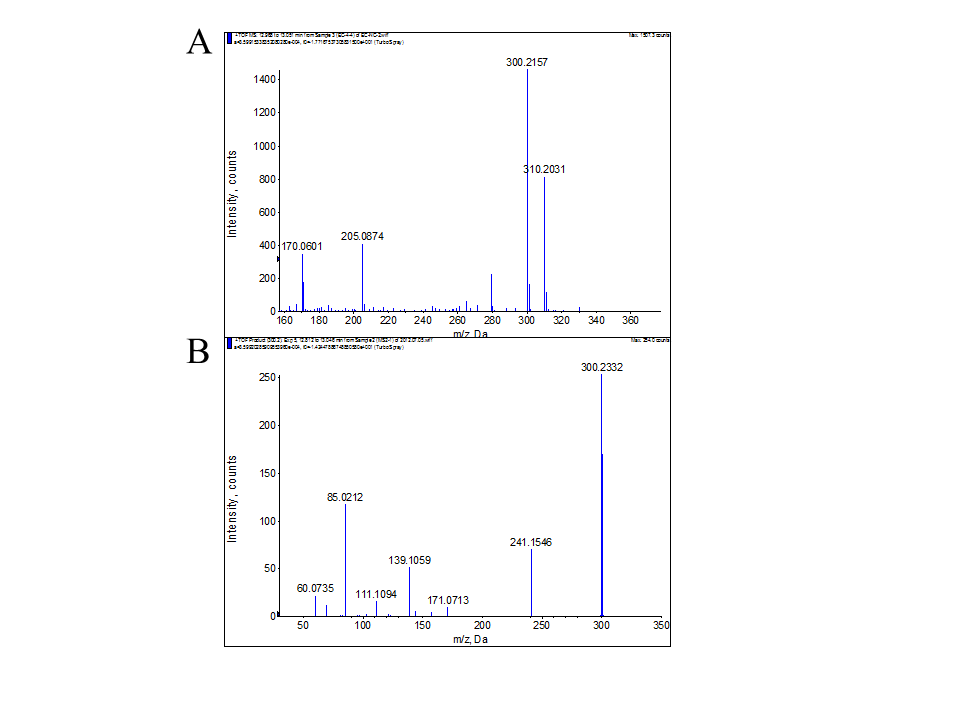


**Figure S20.** MS data used for identification of Nonanoylcarnitine. A: MS spectrum. B: MS/MS spectrum of *m/z* 302.2 (DP=50 V, CE=20 eV).


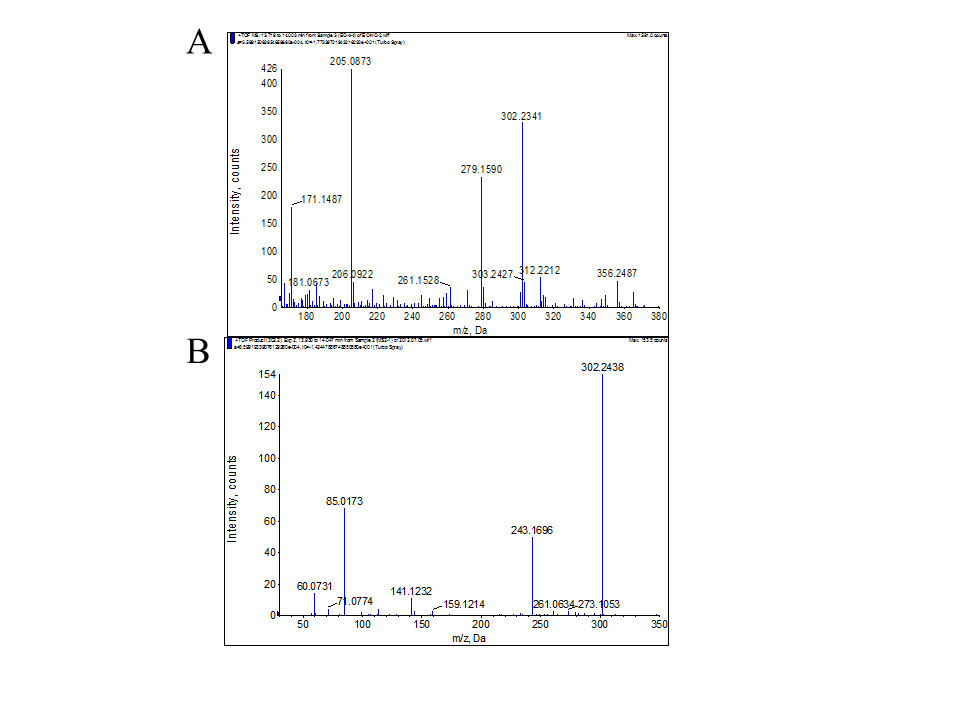


**Figure S21.** MS data used for identification of Decanoylcarnitine. A: MS spectrum. B: MS/MS spectrum of *m/z* 316.2 (DP=50 V, CE=20 eV).


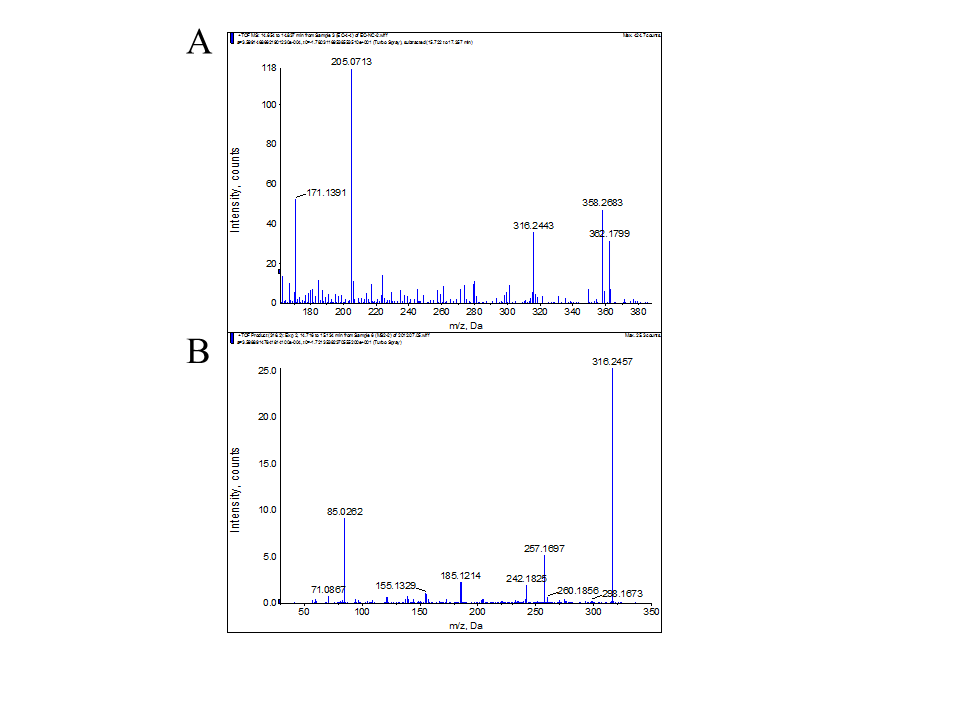


**Figure S22.** MS data used for identification of Undecenoylcarnitine. A: MS spectrum. B: MS/MS spectrum of *m/z* 328.2 (DP=50 V, CE=20 eV).


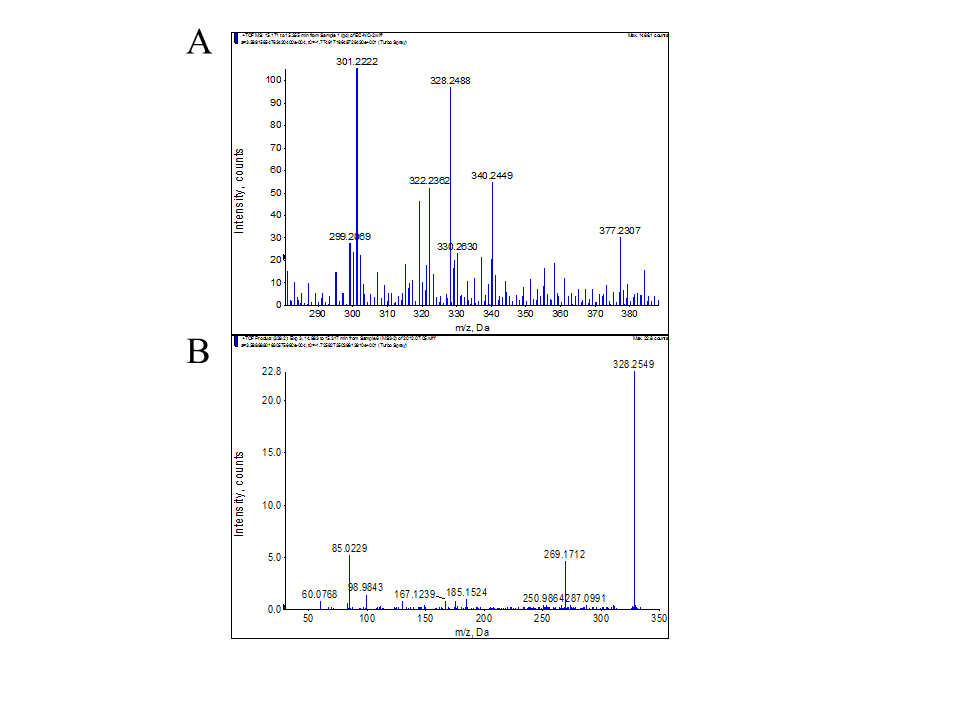


**Figure S23.** MS data used for identification of cAMP. A: MS spectrum. B: MS/MS spectrum of *m/z* 330 (DP=50 V, CE=20 eV).


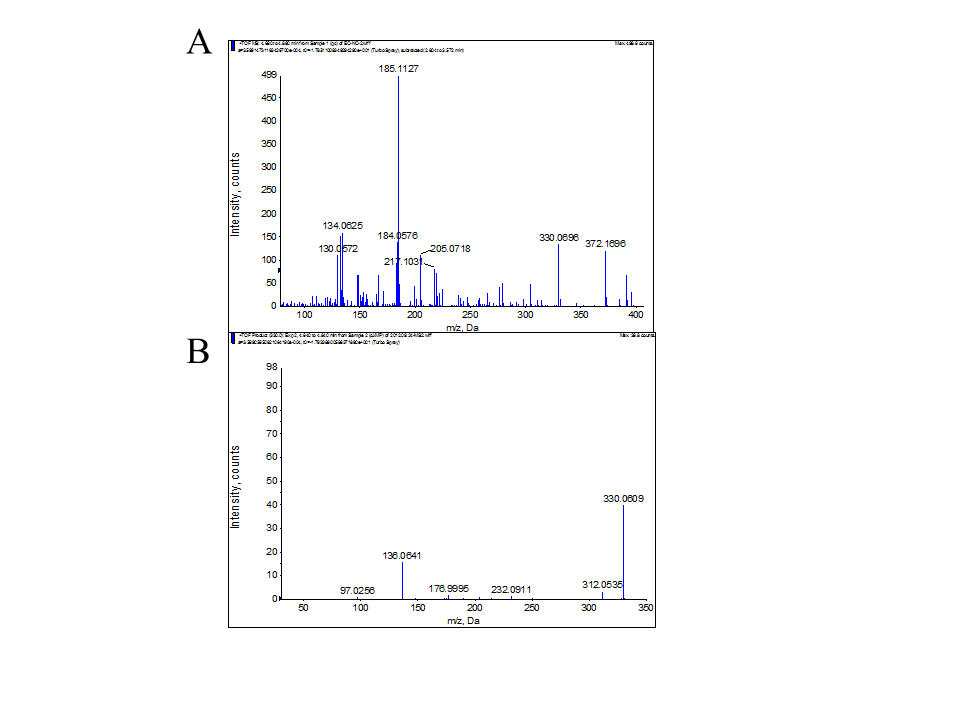


**Figure S24.** MS data used for identification of Undecanoylcarnitine. A: MS spectrum. B: MS/MS spectrum of *m/z* 330.2 (DP=50 V, CE=20 eV).


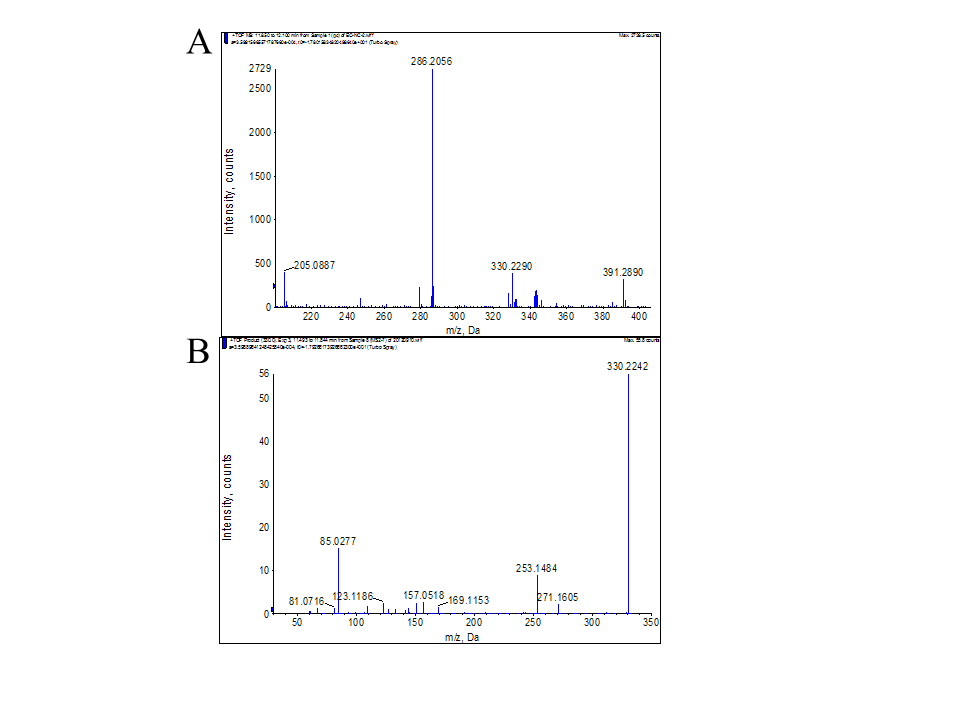


**Figure S25.** MS data used for identification of cGMP. A: MS spectrum. B: MS/MS spectrum of *m/z* 346 (DP=50 V, CE=20 eV).


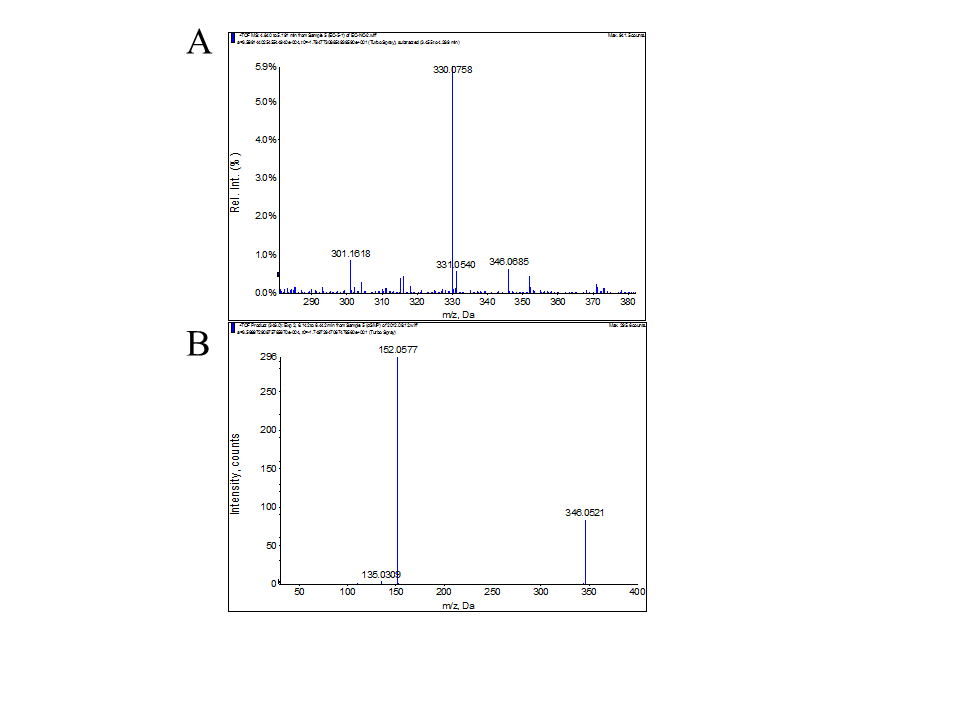

Supplement: Supplementary Information [file srep35010-s1.doc]
